# Adopting the lens of the COM-B behaviour change model to qualitatively explore and understand public health implications of young adults’ attitudes towards death-talk

**DOI:** 10.1186/s12889-025-24519-w

**Published:** 2025-10-02

**Authors:** Catrin Morgan-Duggan, Joanna Brooks, Lisa Graham-Wisener, Christine Rowland

**Affiliations:** 1 Division of Psychology and Mental Health, Faculty of Medicine, Biology and Health, Manchester Centre for Health Psychology, University of Manchester, Manchester, M13 9PL UK; 2 School of Psychology, Queen’s University Belfast, Belfast, BT7 1NN UK

**Keywords:** Public health, Death literacy, Behaviour change, COM-B, Death and dying, Young adults, Advance care planning, ACP

## Abstract

**Background:**

The topic of death and dying holds universal significance, yet societal norms often discourage open discussions, leading to a culture of death-denial. This reluctance can hinder informed decision-making, end-of-life planning, and access to adequate care and grief support. While research has examined death-talk among older adults, clinical populations and healthcare professionals, young adults’ perspectives remain underexplored. Understanding their attitudes is crucial, as early engagement with death-talk – framed within a life-course approach – can foster emotional resilience and contribute to developing compassionate communities. This study aims to explore the perceptions and attitudes of a non-clinical sample of young adults (aged 18–34) towards discussing death and dying.

**Methods:**

A qualitative approach was employed, involving four focus groups with 33 young adults. Participants were selected using maximum variation sampling to ensure diversity in education, ethnicity, gender, and religious beliefs. Reflexive thematic analysis was performed to identify themes related to attitudes toward death-talk, utilising a critical realist stance. Findings were mapped using the Capabilities, Opportunities, Motivations, and Behaviour (COM-B) model of behaviour change, to identify potentially modifiable barriers and facilitators to engaging in death-talk.

**Results:**

The analysis revealed four key themes which collectively illustrate the complex interplay between individual attitudes and broader cultural influences in shaping how young adults perceive and discuss death and dying. The themes highlighted how internal and external factors affect the ability and willingness of young adults to engage in meaningful discussions about death. Factors such as social stigma, fear of causing distress, and a lack of communication skills were identified as significant barriers. Conversely, the recognition of the importance of death-talk, personal experiences with bereavement, and a supportive social environment were found to facilitate these discussions.

**Conclusions:**

This study provides valuable insights into the perceptions and experiences of death-talk amongst young adults, including barriers and facilitators. The findings suggest the need for targeted interventions to enhance death literacy among young people, emphasising the importance of normalising these conversations in everyday life. Recommendations are proposed for utilising these insights to inform public health strategies, education, and policy development aimed at fostering a more open dialogue about death and dying within the broader community.

**Supplementary Information:**

The online version contains supplementary material available at 10.1186/s12889-025-24519-w.

## Background

Globally, the number of deaths is projected to rise to approximately 75 million by 2040, driven largely by population growth and aging demographics [1]. With an ageing population, an increased prevalence of chronic and often complex health conditions [2], and the impact of recent global events, such as the COVID-19 pandemic, the reality of mortality and loss is likely to be increasing in salience across all age groups. Given these factors, whilst an increasing number of people will be impacted by caregiving, death, dying and bereavement, many people with serious illness and their families already lack the support they need, and this gap is likely to widen in future [3]. Palliative care aims to reduce symptoms and treatment effects, and to provide emotional support, practical help, and improve quality of life for people diagnosed with a terminal illness and their carers. The need for palliative care is projected to increase substantially over the next two decades [4, 5]. Although growing evidence supports its effectiveness and cost-effectiveness, palliative care is a neglected health issue globally [6].

High-quality palliative care is underpinned by advance care planning (ACP). ACP is an ongoing process that supports patients in sharing their values, goals and preferences regarding future medical care during serious and chronic illness. A common barrier to good quality ACP is the perception that it is intended for only people at the end of life rather than being about future care planning more generally [7]. Only a minority of adults in the UK have engaged in an ACP conversation [8].

It is generally assumed that people are uncomfortable about engaging in ‘death-talk’ [9, 10]. The promotion of public talk about death and dying has been the focus of various campaigns [11, 12] in the United Kingdom based on the assumption that not doing so is problematic [10]. There have been calls for a public health approach to encourage more open conversations about death and dying [13], with current policy aiming to increase the uptake of ACP [10]. Public health and health promotion initiatives to develop ‘death-literacy’ (the knowledge and skills that make it possible to gain access to, understand and make informed choices about end of life and death care options) can facilitate a whole-community approach to supporting those who are experiencing death, dying and bereavement [14]. This aligns with ‘the new public health approach’ within palliative care [9, 15, 16], which advocates for building community capacity through developing public death literacy and for both community-led and national conversations around death and dying [17]. Community death literacy may help to relieve carer burden, increase engagement with palliative care services and avert potential conflict between different health professionals, family members and care agencies as well as enabling people to better express their end of life wishes [3].

Existing research focusing on perceptions of and attitudes toward discussions around death and dying has primarily targeted clinical populations [18], healthcare professionals [19], and older adults [20]. Previous qualitative studies have focused primarily on the clinical aspects of discussions around death and dying, including palliative care [21] and ACP [22]. Although there has been some work looking at public views of palliative care [23, 24], there has been little research conducted into the general public’s attitudes towards discussion of death and dying. Whilst it is not necessarily always the case that people are reluctant to talk about death [10], research into the attitudes of the general public in the UK indicates that many do report feeling uncomfortable discussing death and dying [25], and actual engagement remains low [13]. Other findings suggest that talk of death is avoided to avoid discomforting others, potentially hindering the initiation of such discussions [26, 27].

Islam et al. [27] conducted an online, mixed methods survey exploring knowledge, attitudes, plans, and preferences towards death and end of life care with 2,210 members of the general public in Wales. Participants reported that social taboos surrounding discussions about death, lack of opportunities and skills to initiate these conversations and personal fear and discomfort hindered open dialogue about death and dying. Graham-Wisener et al. [9] replicated Islam et al.’s [27] work in Northern Ireland. The analysis revealed a range of barriers and facilitators to death-talk. Themes included the lack of social acceptance of death, fear of causing distress and the need to enhance communication skills to facilitate conversations. Both studies lacked a dedicated focus on young adults among their participants: in Islam et al.’s [27] survey, 11.5% of respondents were aged 18–34, and in Graham-Wisener et al.’s [9] study, 22.6% were younger adults. Both studies also reported underrepresentation amongst younger adults who did take part in terms of intersectional factors such as socioeconomic status, ethnicity and gender. A representative survey of the general public in Germany [28] identified characteristics including gender, age, religion, and socioeconomic status associated with different attitudes towards talking about death and dying. Men, younger adults and those who did not own their own homes were less likely to engage in death-talk. There is then some preliminary evidence suggesting that particular groups may be less likely to engage in death-talk than other demographics, but also that these groups are less likely to be included in research in this area. To effectively develop public death literacy and community-led conversations around death and dying, research needs to be undertaken with complete communities including ‘hidden publics’ [15]. There have been recent calls in the literature for a greater focus on young adults in the context of research into discussions around palliative care, death and dying [29, 30]. Although there has been work to develop initiatives to facilitate communication with children about death and dying [31, 32] and calls to integrate teaching about death and bereavement into school curriculums [33, 34], there is much less work undertaken with young adults in this area. This is acknowledged by recent calls in the literature for a greater focus on young adults in research into discussions around palliative care, death and dying [29, 30]. Work undertaken to explore young adults’ understanding of palliative care specifically [21] highlights the need for young adults’ input into the development of any community public health initiatives to develop death literacy and support whole community approaches to death, dying and bereavement.

**Fig. 1:**
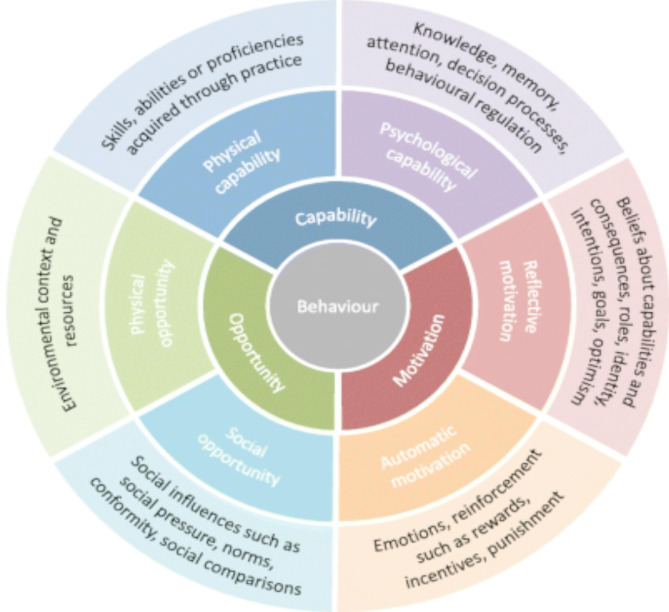
The COM-B model and components [39]

To develop interventions to increase death-talk at a population level also requires a theoretically informed understanding of the target behaviour and change processes associated with it [9, 35]. There is limited but increasing use of theoretically informed interventions and behaviour change theory within palliative care research [9]. In their examination of public talk about death and dying in Northern Ireland, Graham-Wisener et al. [9] mapped barriers and facilitators identified onto health behaviour change models used in public health: the Behaviour Change Wheel [BCW] [36] and the Theoretical Domains Framework [TDF] [37]. The COM-B [36] (see Fig. 1) forms part of the BCW and identifies determinants of behaviour (B) in terms of capability to perform the behaviour (C), opportunity to engage in the behaviour (O) and motivation to undertake the behaviour at a given time (M). The Theoretical Domains Framework (TDF) consists of 14 constructs which are mapped onto the COM-B model in order to further analyse the determinants of behaviours [38]. Graham-Wisener et al. [9] present a theoretical understanding of the drivers of death-talk, with barriers and facilitators to talking about death and dying mapped to the majority of the COM-B and TDF components but conclude that more research is needed to confirm if these drivers are relevant for the wider population and ‘hidden publics’ in particular.

With few exceptions [40] there is limited research examining barriers and facilitators to talking about death and dying in general population samples, with especially little work currently undertaken to facilitate more in-depth responses. This is the first study to examine exclusively barriers and facilitators to death-talk in young adults and one of a small number of studies to apply health behaviour change theory to this topic. To address this and extend the previous work undertaken by Graham-Wisener et al. [9], this study aims to (1) explore how younger adults perceive and approach conversations about death and dying and (2) use behaviour change theory (COM-B) to identify potentially modifiable barriers and facilitators to engaging in death-talk amongst this group.

## Methodology

We have drawn on the guidance provided in the Journal for Reporting Standard for Qualitative Research (JARS-Qual) [41] for describing the study methodology.

### Methodological approach

This was a cross-sectional, qualitative focus group study using a semi-structured topic guide, approved by the University of Manchester Ethics Committee (reference 2024-18667-32792). The study was underpinned by a critical realist theoretical framework which acknowledges an external reality at the ontological level, but recognises that epistemologically, our knowledge of this reality is shaped by our social, cultural and individual contexts. This position is congruent with the approach adopted in this study, which sought to explore subjective meanings and lived experiences. Taking a critical realist perspective, the analysis aimed to interpret young adults’ perceptions of and attitudes towards discussing death and dying, with the goal of identifying patterns of meaning that reflect situated truths expressed in the dataset [42].

Focus groups were selected as a method consistent with the study’s critical realist and qualitative orientation, enabling the exploration of how young adults collectively make sense of societal attitudes towards death and dying. Rather than focusing on individual narratives or experiences, the study aimed to investigate shared understandings and social processes, making focus groups especially suitable. The group setting encouraged participants to engage with one another’s views, which supported the expression of social norms, the questioning of assumptions, and deeper reflection on how death is discussed in society—an approach particularly well-suited to the study’s focus on discourse around death. Virtual focus groups were conducted to facilitate participation across different regions of the UK and to offer a flexible, accessible format for a geographically dispersed sample.

### Sampling and recruitment

A maximum variation purposive sample targeting a range of factors found in previous research to influence engagement in death-talk (education level, age, ethnicity, gender, and religious beliefs) was adopted [28]. This study’s target population was young adults between 18 and 34. The maximum age of 34 is aligned with the age categories established within the existing literature [9, 27], allowing for ease of comparison across studies and results. Participants were required to complete an open-response demographic questionnaire hosted in Qualtrics before group allocation. Inclusion criteria were: between 18 and 34 years of age, proficient in spoken English language and access to an internet-capable device. There were no exclusion criteria for participation.

Participants were recruited through an advert posted online via social media and in three geographical locations in Wales and England (e.g. posters in cafes). Those interested were sent an information sheet, link to demographics questionnaire, and provided informed consent before participation. At all stages participants were reminded that the subject of the research was death and dying, and were asked to consider if they felt able to participate without causing themselves distress.

Of 46 potential participants who expressed an interest in the research, 33 consented to take part. Reasons for non-participation included schedule unavailability for proposed groups and reflection that the topic under discussion may cause distress or discomfort. Given the non-anonymous nature of the focus group setting, a comprehensive distress protocol was in place: participants were monitored for signs of distress, offered breaks or the option to withdraw at any time, and provided with detailed information about university and external support services in the form of a debrief sheet provided via email.

### Data collection

Four focus groups of between five and ten participants each were held virtually via Zoom. Groups lasted an average of 70 min. Audio recordings were made, transcribed verbatim and pseudonymized. Data collection was carried out by the first author (CMD) and overseen by the last author (CR).

An extensive literature review, including the prior research paper by Graham-Wisener et al. [9], informed the topic guide used to facilitate group discussion. The topic guide included seven main questions, with between 1 and 4 nested sub-questions and was co-designed by two researchers (CMD, CR) and reviewed by a third (JB). Topics included social acceptability and comfort with death-talk, its perceived importance, changes over time in how death-talk is approached, and suggestions for overcoming barriers and enhancing facilitators to these discussions. All included questions stressed a general perspective, rather than asking about personal experiences.

Given the non-anonymous nature of the focus group setting, a comprehensive distress protocol was in place: participants were monitored for signs of distress, offered breaks or the option to withdraw at any time, and provided with detailed information about university and external support services in the form of a debrief sheet provided via email.

### Analysis

Reflexive thematic analysis (RTA) [43] was conducted. This approach aligns with the critical realist perspective that underpins the research by recognising that while individuals’ interpretations of the world are inherently subjective, these interpretations are shaped by broader social structures.

The six-stage process of RTA outlined by Braun and Clarke [42] guided the analysis. Analysis began with repeated reading of transcripts to support familiarisation with the data. This was followed by systematic inductive coding of segments deemed meaningful or relevant to participants’ beliefs and experiences in relation to the research question. Coding was iterative and interpretative, and culminated in the development of initial candidate themes that reflected both semantic and latent patterns. Nine candidate themes were initially identified. These were refined through team discussion - some merged, others separated - ultimately resulting in four final themes that captured a coherent analytic narrative of young adults’ perspectives on engaging in death-talk. Individual codes were collated into sub-themes to represent meaningful patterns and highlight nuanced variations within each main theme. The analysis followed a two-stage process: first, inductive theme development grounded in the data, and second, a theory-informed phase that considered how findings could be situated within broader conceptual frameworks. This approach aligns with the critical realist emphasis on uncovering underlying mechanisms while remaining grounded in participants’ accounts.

Analysis was led by CMD (they also conducted the groups). They are a female postgraduate Psychology student within the target sample age. They have worked in palliative care and have personal experience with the deaths of close family members. CR is a female lecturer in psychology and has worked in end-of-life research for over a decade. JB is a female senior lecturer in psychology specialising in qualitative research methods, with personal and professional experience of end of life and palliative care. LGW is a female reader in psychology and has worked in palliative and end-of-life care research since 2016.

Reflexivity was central to analysis and involved continuous questioning of our interpretations, considering alternative explanations, and engaging with both individual accounts and the wider contexts in which they were situated. While full understanding of participants’ experiences is always partial, some experiences and meanings can be seen as more widely shared, forming a degree of objectivity at the contextual or conceptual level. A reflexive diary was used by the first author (CMD) to help consider how their beliefs and experiences influenced data collection and interpretation.

Reflexive practice extended beyond individual reflection and was supported by regular team discussions that encouraged critical dialogue, challenged assumptions, and contributed to the development of rigorous and well-considered interpretations. These discussions also served to make visible how our own positionalities may have shaped the analytic process, helping to navigate the interplay between subjectivity and the identification of shared patterns in the data.

In a separate analytical phase, key barriers and facilitators to engaging in death-talk were mapped onto the COM-B model [36], where they were conceptually aligned with the domains of capability, opportunity, and motivation [9]. This mapping process involved developing descriptions derived from created themes and codes, which aligned with the corresponding conceptual components of the COM-B.

The decision to apply the COM-B framework was made a priori, as outlined in the introduction. Importantly, this mapping occurred after the completion of inductive thematic analysis, allowing themes to emerge organically before being considered in relation to the framework. This pragmatic approach combined the strengths of data-driven analysis with the explanatory utility of an established model for understanding behaviour and behaviour change. This final phase permitted key findings to be articulated in clear, accessible terms, supporting interpretation, enhancing the reach of the research and the practical relevance of the results.

## Results

Participant demographics are presented in Table 1. There were similar proportions of males and females (51.5%; 48.5% respectively).The majority of participants were aged 18–25 years (63.6%) and were of a white ethnic group (54.6%). Almost half identified as non-religious (45.5%) and had attended higher education (57.6%).

**Table 1:** Participant demographic characteristics (*n *= 33)

	*n*	%
Gender		
Female	17	51.5
Male	16	48.5
Religion		
Christian	3	9.1
Muslim	3	9.1
Atheist/None	15	45.5
Agnostic	7	21.2
Other	5	15.2
Ethnicity		
White British	15	45.5
Asian British	9	27.3
Black British	6	18.2
White European	3	9.1
Age		
18-21	8	24.2
22-25	13	39.4
26-29	5	15.2
30-34	7	21.2
Highest educational level		
Secondary (GCSE/Level 2)	3	9.1
Further (A- level/Level 3)	11	33.3
Undergraduate	10	30.3
Postgraduate	9	27.3

Percentages may not total 100.0 due to rounding

### Study Aim 1: to explore how younger adults perceive and approach conversations about death and dying

Four themes which encapsulate young adults’ perceptions of and attitudes towards discussing death and dying, are presented in Table 2: ‘Obscuring Content of death-talk’, ‘Barriers to death-talk’, ‘Media Shaping death-talk’ and ‘Death-talk as a Skill’.

**Table 2:** Thematic table

Theme	Sub-themes	Supporting extracts
Obscuring content of death talk	✥ Making content more palatable	‘*When you talk about death and dying... it's always quite general like you'd say, oh, they passed away rather than - people very much avoid talking about how they died or the circumstances around their death’*
	✥ Use of humour	‘*British humour, quite like - black humour, it’s very much kind of make a joke about it rather than cope with the real thing’*
	✥ The problem with platitudes	*‘The last thing you want to hear is just like aw there there or it’ll be ok * *you know, just like generic sort of, pat on your shoulder sort of thing…* * its being able to open up and have a more in-depth conversation than just a polite sort of passing of the conversation’*
Barriers to death talk	✥ Fear of causing distress	*‘I would feel, especially if the person became upset, that I’d caused that upset’*
	✥ Existentialism: Fear of own mortality	*‘Talking about death is going to lead you to like thinking about or talking about your own death. And I think that's something that not many people or quite a few people, aren't willing to confront or like think about’*
	✥ Wholly negative perceptions of death	*‘In society its seen as something really sad, isn’t it? And people don’t really want to talk about that’*
Media shaping death talk	✥ Exposure to extreme cases	*‘I think a lot of the time when we see death, especially on the news, it's always a very like traumatic event that's happened’*
	✥ Installation of anxiety	*‘It comes to a point where it's affecting you, and it makes you so anxious that you just want to isolate yourself’*
	✥ Statistical numbing and dehumanisation	*‘It can be quite difficult to imagine that every single one of those numbers is a person… and it’s kind of disassociated people from… You know, from death’*
Death talk as a skill	✥ Uncertainty around supporting others	‘*You want to comfort them, but you don’t want to upset them’*
	✥ Need for instruction	*‘We’ve never really been... taught how to approach those conversations’*
	✥ Promoting early conversations	*“I think if in schools they put like sections aside... that would open an opportunity for discussions to be had at a younger age… and people would be more comfortable sharing experiences’*

#### Obscuring content of death-talk

This theme captures the various ways in which individuals navigate their discomfort around discussing death and dying. Rather than engaging directly with the subject, participants discussed employing strategies that obscure the explicit details of death, aiming instead to make the topic (as they perceive it) more palatable and socially acceptable. These mechanisms, such as humour and masking, serve to distance individuals from the emotional intensity of death, allowing them to discuss the subject without becoming overwhelmed by its implications.

#### Making content more palatable

Young adults sought to discuss death in a way they considered to be less distressing and more acceptable in social contexts. This involved using euphemisms, avoiding explicit details of end-of-life, and redirecting the conversation to focus on what they perceived to be less threatening aspects. These strategies help individuals manage the discomfort associated with death-talk and navigate social norms that render death a taboo subject for some.

‘*When you talk about death and dying… it’s always quite general like you’d say*,* oh*,* they passed away rather than - people very much avoid talking about how they died or the circumstances around their death’ (Emily*,* 21).*

By keeping discussions general, participants can engage with the topic while avoiding specific details, presumably making it easier and more acceptable to discuss in social settings.

*‘When someone’s died*,* we’ll talk about their life rather than actually dying. They ‘ll tell like stories of when they did this*,* or something happened*,* that like avoids the fact that they died’ (Anna*,* 18).*

The quote from Anna illustrates the importance of storytelling in the process of grieving. However, Anna also notes a particular strategy of shifting the focus from the specific event of death itself to the life and stories of the deceased. This approach may help manage the discomfort associated with discussing death by allowing individuals to celebrate the person’s personality and memorable moments, keeping the conversation positive and less emotionally taxing. The phrase “*avoids the fact that they died*” highlights a deliberate omission of the death event in discussions, suggesting a reluctance to engage with the realities of death. Individuals can maintain a sense of normalcy and emotional equilibrium by steering the conversation away from the details of dying.

#### Use of humour

Humour was a common strategy employed by young adults to make conversations about death more approachable. Humour served as a tool to alleviate discomfort, foster connection, and reduce the emotional weight of a conversation.

‘*British humour*,* quite like - black humour*,* it’s very much kind of make a joke about it rather than cope with the real thing’ (Risha*,* 21)*.

‘*From my work as a nursing assistant in the hospital*,* we always kind of had a bit of dark humour to deal with it’ (Karl*,* 33)*.

Using humour helps to break down barriers which typically surround death-talk and allows for more open dialogue.

#### The problem with platitudes

In addition to humour, participants reported shifting conversations about death towards positive aspects, focusing on discussing positive memories or humanistic philosophies.

*“If they’re talking about it*,* they tend to drift into a positive thing and talk about like the value of life. And that’s almost avoiding the topic of death” (Katie*,* 26)*.

Whilst reframing can feel helpful, it can also result in diminishing or dismissing a person’s experience and desire to discuss the topic of death itself. This can engender a superficial engagement with the subject, preventing deeper and more beneficial explorations of grief and loss.

*‘The last thing you want to hear is just like ‘aw there*,* there’ or ‘it’ll be ok’*,* you know*,* just like generic pat on your shoulder sort of thing… it’s being able to open up and have a more in-depth conversation’ (Simon*,* 31)*.

Whilst the type of platitudes reported by Simon, and strategies of obscuring, masking, and reframing discussions around death serve to make the topic more socially acceptable and emotionally manageable, they may have unintended consequences. Such approaches can be used with the intent to help individuals navigate discomfort, maintain a sense of normalcy and even offer temporary relief from grief. However, they may ultimately prevent deeper engagement with the reality of death, fully processing grief and exploring the complexities of loss.

#### Barriers to death-talk

This theme captures the various psychological and emotional barriers that young adults face around initiating and engaging in death-talk. Participants discussed barriers stemming from deep-seated anxieties shaped by personal, cultural, and social influences. For many, discussing death evokes discomfort, uncertainty, and emotional vulnerability, leading to a reluctance to broach the subject. Furthermore, these barriers reflect broader societal attitudes around death, where silence or avoidance is often the norm.

#### Fear of causing distress

Participants were reluctant to engage in conversations about death due to the fear of causing emotional distress to others, such as provoking tears or heightened grief. This, in turn, generated feelings of guilt or responsibility for any upset caused rather than merely a natural, linked expression of grief or sadness by the individual.

*‘I would feel*,* especially if the person became upset*,* that I’d caused that upset’ (Simon*,* 31)*.

This apprehension is compounded by a cultural tendency to avoid awkward or uncomfortable conversations and situations, which some attribute to a broader societal norm of maintaining superficial harmony.

*‘We’re just a nation of people pleasers. So*,* if we can get along with just being happy*,* not address anything awkward*,* I think that’s just what we’re going to do’ (Cara*,* 32)*.

#### Existentialism; fear of own mortality

Underpinning the apprehension about causing upset to others is a sense of the existential fear associated with death, which creates a significant barrier to engaging in death-talk. Discussing death inevitably led participants to confront their own mortality, which many found deeply unsettling.

*“Talking about death is going to lead you to like thinking about or talking about your own death. And I think that’s something that not many people or quite a few people aren’t willing to confront or like think about"​ (Olivia*,* 21)*.

The dread of death triggers existential questions about life’s purpose and what happens after death, leading to avoidance.

*‘A lot of people bury their head in the sand when it comes to their own death because they don’t want to think about it… it brings up a lot of emotions*,* and they think*,* what am I doing with my life sort of thing’ (Matty*,* 25).*

#### Wholly negative perceptions of death

Participants perceived death in an overwhelmingly negative light, describing societal norms that cast death as inherently sad and depressing, devoid of any positive aspects, further inhibiting death-talk.

*‘‘In society*,* it’s seen as something really sad*,* isn’t it? And people don’t really want to talk about that’ (Kane*,* 28).*

Some participants suggested that this negative perception is reinforced by a lack of available cultural practices that celebrate or normalise death, as in other cultures.

*‘there’s not like processes like the day of the dead that will make it like a fun thing. It’s just like a just - just a sad thing. So*,* people don’t really want to talk about sad things’ (Eve*,* 25).*

*‘I think we just don’t celebrate it like in - like other places in the world actually have little celebrations for like dying and death’ (Lily*,* 34).*

This theme highlights how personal fears, and cultural norms create psychological barriers for young adults when discussing death. The lack of cultural practices and tools prevent the normalisation of death-talk and creation pathways to invite conversations about death at a community level. Such barriers, while serving to protect individuals from discomfort in the short term, hinder open discussions and broader societal acceptance of death. Fostering greater openness, knowledge, and acceptance could be beneficial in helping young people develop resilience and gain meaningful insights into life and mortality, ultimately shifting perceptions of death away from being ‘wholly negative’.

#### Media shaping death-talk

The media is an important source of communication around death and dying for young adults, and how it is discussed can impact their beliefs, perceptions and attitudes around engaging in death-talk. Participants’ responses highlighted the pervasive impact of media representations. Extreme and often sensationalised or sanitised portrayals of death contribute to a skewed understanding and a heightened sense of fear and detachment. Overall, participants felt that the media impacted how they perceived the appropriateness of discussing death in everyday life and, importantly, the way this should be done.

#### Exposure to extreme cases

Exposure to extreme cases of death through media influenced participants’ perceptions and discussions. Participants frequently mentioned how the media highlights traumatic and violent deaths, which can skew public perception towards viewing death in predominantly distressing and negative ways.

*‘I think they talk more about death in terms of genocides and wars that are happening’ (Lauren*,* 23)*.

*“I think a lot of the time when we see death*,* especially on the news*,* it’s always a very like traumatic event that’s happened"​ (Selina*,* 22)*.

This exposure to extreme cases can desensitise people to death or make it a topic associated primarily with fear and negativity.

*‘It’s not uncommon to hear like 6-year-olds talking about watching beheadings or shooting games or videos of actual mass shootings like horrific things because they are on YouTube’ (Jane*,* 27.)*

This desensitisation suggests an interesting dichotomy whereby exposure should perhaps make death and dying easier to discuss, but the extreme nature and distance from the events and persons involved lead to desensitisation and distancing. Where discussion is at the individual level and relating to someone known to participants, desensitisation did not appear to occur.

#### Instillation of anxiety

Some participants experienced significant anxiety at what they saw as a result of the media narrative around death; specifically coverage of fatal events. Anxiety was felt to be heightened even further when the emotional connection was made between sensationalised media reported deaths and the context of their own lives.

*‘Stuff like this that’s going on in the news all the time and online articles like I think there is a sort of foreboding air to the world at the moment that a lot of people are feeling*,* and that is giving a lot of people anxiety’ (Sam*,* 30)*.

This continuous barrage of negative and fear-inducing information led people to avoid media coverage of events and discussing death.

*‘It comes to a point where it’s affecting you*,* and it makes you so anxious that you just want to isolate yourself’ (Lauren*,* 23)*.

Extreme media coverage of death which induces anxiety in these young people, may lead them to avoid the topic altogether or refrain from discussing it with others. This, in turn, reinforces the societally entrenched belief that death is a subject to be avoided.

#### Statistical numbing and dehumanisation

The quantification of death through a focus on death tolls and body counts in the media resulted in participants feeling removed from the lived reality of events and dehumanisation of the deceased. Examples of this included coverage of events like the COVID-19 pandemic.

*‘It can be quite difficult to imagine that every single one of those numbers is a person… and it’s kind of disassociated people from… You know*,* from death’ (Ben*,* 19)*.

This numerical representation can create a disconnect, making it difficult for people to empathise with the human impact behind the numbers, further desensitising them to death and diminishing the emotional weight typically associated with such loss. Overall, this theme covers the nuanced ways in which media narratives shape death-talk perspectives amongst young adults and are related to personal anxieties and pervasive beliefs around the wholly and inherent negativity of the subject.

#### Death-talk as a skill

This theme underscores the perspective amongst young adults that discussing death is a learned ability requiring guidance and practice. Participants expressed a desire for direction on initiating and sustaining effective and meaningful discussions, reflecting a perceived gap in educational curricula and support in navigating these conversations. Engaging in death-talk was not seen as an innate skill but rather one that could be developed through instruction and experience.

#### Uncertainty around supporting others

Participants expressed an instinctive desire to provide comfort but reported that this is often hindered by uncertainty about the appropriate actions. This instinctive need clashed with the fear of causing additional distress, making it challenging to offer meaningful support.

‘*You want to comfort them*,* but you don’t want to upset them’ (Simon*,* 31).*

This uncertainty often stems from a fear of saying the wrong thing or exacerbating the griever’s pain.

*“It becomes such an uncomfortable topic because people don’t know how to respond to it maybe or how to just help somebody going through a bereavement"​ (Lauren*,* 23).*

This suggests that in feeling responsible for causing upset, as explored in a previous theme, at least some young adults are motivated to learn how to navigate these conversations sensitively and provide support for those engaged in them, rather than avoid them completely.

#### Need for instruction

Participant discussions frequently expressed a need for formal instruction on engaging in death-talk. Many expressed a need for guidance and education on the subject, indicating that although participants consider engaging in death-talk to be a skill, it is not believed to be an innate one.

*“We’ve never really been… taught how to approach those conversations” (Rachel*,* 20)*.

Participants discussed that educational systems often focus on the beginning of life but neglect the end, adding to their feelings of being unprepared for such discussions.

*‘‘I think we could start off by*,* like*,* maybe educating people a bit better on death because*,* like*,* when you go through school and stuff*,* you learn a lot about like new life… But there isn’t really anything about death and bereavement (Rob*,* 23).*

Participants also implicated the perceived lack of institutional guidance around emotional coping and wider life skills in leaving young people feeling unprepared and unequipped to engage in discussions around death and dying.

*‘‘We have all said throughout this discussion that we’re not really taught the life skills’ (Katie*,* 26).*

#### Promoting early conversations

Participants advocated strongly for the introduction of death-talk education at a younger age. Participants believed that early education could normalise conversations about death and equip young people with the necessary skills to handle bereavement later in life.

*“I think if in schools they put like sections aside… that would open an opportunity for discussions to be had at a younger age… and people would be more comfortable sharing experiences” (Katie*,* 26).*

*‘Just educating people*,* sort of normalising death and then obviously that could better equip young people to*,* you know*,* support themselves and others through bereavement later in life’ (Rob*,* 23).*

This approach was seen as a way to foster openness and honesty about death from an early age, thereby reducing the fear and stigma associated with it. Participants suggested that often children are shielded from such topics, but this is unnecessary and potentially detrimental.

*‘It’s something that adults know about. It’s too sad for children to know – then that just instils in us from a really young age*,* that it’s like something to be really scared of’ (Jane*,* 27).*

This theme reveals the inherent perceptual complexities, discomfort, and uncertainty associated with offering emotional support in the context of bereavement. Further, it highlights the gap in educational systems, which often neglect to equip individuals with the skills necessary for navigating death and bereavement. In addressing this, young adults could be better equipped to navigate such sensitive topics and better support others in loss.

### Study Aim 2: identify potentially modifiable barriers and facilitators to engaging in death-talk, using COM-B

The barriers and facilitators to discussing death and dying, which emerged through participant discourse, were mapped onto constructs within the COM-B model where they were deemed to conceptually fit.

Considering *psychological capability* (see Table 3), participants identified explanations of avoiding death-talk based on limiting psychological beliefs that they lack capability for these conversations and the discomfort associated with them. Facilitators included normalising public and educational discourse around death and dying.

**Table 3:** Barriers and facilitators mapped to the behaviour change wheel COM-B

COM-B Component	Barriers	Facilitators
Psychological Capability	✥ Having a lack of experience around death makes engaging in a supportive or helpful way more difficult	✥ Hearing about people’s lived experiences around death and dying – beyond the extreme, dramatic instances reported in the news, would help to normalise the topic
	✥ The idea that specific skills are needed to engage in death-talk, and that these are not taught and so not embodied, leading to avoidance due to fear of doing or saying the ‘wrong’ thing	✥ Encouraging discussions about death and dying in early years, as well as insights around how to open these conversations in people’s own lives, would work to increase comfortability with engaging in death-talk and destigmatise the subject
		✥ Teaching skills around how to converse around emotive topics, and to provide support to those impacted by death and dying
Physical Capability	-	-
Social Opportunity	✥ There is a cultural emphasis on privacy in the UK, which hinders people’s ability to share their experiences or engage in death-talk	✥ Knowing of people’s similar or shared experiences around death and dying increases comfort as well as perceived acceptability of engaging in death-talk
	✥ Individualized societies have a disconnect whereby social opportunities to learn of, and therefore to engage in discussion around, deaths that have occurred is limited	✥ Ceremonies such as funerals and wakes help to facilitate conversations around death and dying amongst social circles by bringing people together and creating a sense of collective grief
	✥ Having strong, emotionally connected relationships with family and friends can make broaching the topic of death-talk more constructive and positive	
Physical Opportunity	✥ Feeling that there is a lack of spaces which facilitate engaging in death-talk	✥ Religious traditions around death, and living in a smaller, collectivist culture allows death-talk to occur more naturally and provides set spaces in which to do so
	✥ Conversations around death and dying tend to take place as it becomes immediately relevant, and this issue around timing during an often emotional period is a challenge	✥ Encouraging celebratory practices around death and dying, community spaces for discussion and positive reflection would facilitate more physical opportunity to engage in death-talk in a beneficial way
Reflective Motivation	✥ The belief that engaging in death-talk will result in emotional and/or social discomfort for those involved	✥ Encouraging conversations around death in the public domain which facilitate an understanding that death is not necessarily always entirely negative, and encourage a more positive cultural attitude
	✥ The belief that death-talk darkens the mood in social settings, and is an entirely negative subject enforcing the assumption that others generally do not want to discuss death and dying	✥ Providing guidance on how to open, and engage in, conversations around death and dying to encourage individuals to feel more comfortable in instigating these discussions
	✥ Feeling unable to discuss death and dying in the ‘right’ – constructive, positive, helpful – way, related to beliefs surrounding lacking skills	
	✥ Engaging in death-talk reminds individuals of their own mortality, and of the discomfort which comes with the unknowable truths around death	
Automatic Motivation	✥ Anticipated negative emotional reactions from others leads to death-talk not taking place	✥ As with mental health, work to destigmatise discussing important experiences, however uncomfortable we may perceive them to be
	✥ Feeling that there is uncomfortable vulnerability in opening up about our own experiences around death and dying	

*Social opportunity* was highly relevant, with participants focussing on beliefs in restrictive social norms that emphasise privacy and provide limited opportunities for learning about and discussing experiences of death and dying in social encounters. Facilitators described the benefits of social occasions that permit acknowledgement and engagement with collective grief by providing opportunities to share experiences around death and dying and create a sense of social unity.

Related to *physical opportunity* to engage in death-talk, a lack of pre-emptive opportunities to engage in death-talk and space to conduct these conversations were highlighted as substantial barriers. Participants recognised the increased physical opportunity to engage in conversations in religious groups and smaller collectivist cultures and contexts could facilitate engagement in death-talk. Further, participants expressed beliefs that encouraging celebratory practices around death and dying, community spaces for discussion and positive reflection would facilitate more physical opportunities to discuss death and dying.

In terms of barriers related to *reflective motivation*, participants discussed several constraining beliefs, for example, the emotional impact death-talk can have on others, or believing it can negatively influence the mood in social settings, the view that death is an entirely negative subject and feeling unable to discuss death and dying in the “right” way. Facilitators included a public discourse approach to encourage a more positive cultural attitude, and guidance on engaging in conversations around death and dying.

Considering *automatic motivation*, the anticipated negative emotional reactions from others and the feeling that there is an uncomfortable vulnerability in opening up about personal experiences were identified as barriers to discussing death and dying. Facilitators focused on strategies for encouraging a shift in social beliefs through formal skills education programmes, such as how to converse around emotive topics and provide support to those impacted by death and dying. Further, destigmatising honest discussion of human experiences more generally (.e.g., mental health), however uncomfortable they are perceived to be, could further facilitate willingness engage in death-talk.

*Physical capability* was identified as the least relevant COM-B construct for the participants in this study who did not discuss any physical limitations which may impact their ability to engage in death-talk.

## Discussion

This study contributes to a growing evidence base exploring death-literacy as a public health issue. To our knowledge we are the first to report exclusively on the perceptions of and attitudes towards engaging in death-talk amongst young adults and interpret this through a behaviour-change model (COM-B). Using qualitative methods, four themes were identified which collectively illustrate the complex and multifaceted nature of death-talk among young adults, revealing significant barriers and opportunities for enhancing these critical conversations which are understood within the themes: ‘Obscuring the Content of Death-talk’, ‘Psychological Barriers’, ‘Media shaping Death-talk’ and ‘Death-talk as a Skill’. The use of focus groups permitted more in-depth exploration of the wider perceptions and attitudes around death-talk, providing context for identified barriers and facilitators [9] and has done so with a previously under-researched group.

By situating this research’s findings against the literature that has informed it [9, 27], we have gained novel insights, including the knowledge that young people think similarly to older adults in UK regions. This previous work identified how feelings of inappropriateness [27] and the unacceptability of death-talk in social contexts [9] hindered conversations. Our work has developed a greater depth of understanding of this. Our theme, ‘Obscuring Content of death-talk’, captures new information about how young people manage and engage in these conversations, given this perceived unacceptability and inappropriateness. The theme ‘Death-talk as a skill’, confirms findings from other population groups which advocate for formal education around death and for starting these conversations at an early age [27] to account for apprehension around navigating these topics [9]. While this work supports some of the narratives developed in earlier studies, it focuses more on the intricacies of engaging in death-talk rather than on broader clinical considerations such as knowledge and preferences around end-of-life care [27].

Analysis using the COM-B framework provided valuable insights into the barriers and facilitators to death-talk among young adults. Key findings highlighted psychological capability barriers, such as perceived inadequacy and discomfort, which could be mitigated by normalising death discussions through education and public discourse. Social and physical opportunities were limited by restrictive social norms and a lack of appropriate spaces, suggesting that creating communal spaces for these conversations (for example, a ‘before I die wall’ introduced within spaces routinely occupied by young people) could enhance engagement. Reflective and automatic motivations revealed that negative perceptions and emotional reactions hindered open dialogue, indicating a need for destigmatisation and emotional literacy to foster comfort with these discussions. Importantly, these insights align well with the barriers and facilitators elucidated in previous work [9] and indicate that public health campaigns based on this information can be effective throughout the life course, rather than necessarily requiring different approaches. As such, there is a need to ensure that any such initiatives are accessible and acceptable to younger adults as well as other demographic groups.

Our analysis highlighted participant tendencies to avoid direct discussions about death by employing a range of strategies which serve to obscure explicit details and make conversations perceived to be more socially acceptable. Whilst guidelines emphasise the importance of clear communication when discussing death, hesitancy to reference death in explicit, unambiguous terms remains dominant [44]. It has been noted that ‘the language of death’ has been lost and that this is an increasingly common phenomenon [45]. A departure from explicit language choices and towards implementing obscuring strategies may serve as a coping mechanism, allowing individuals to maintain emotional comfort. However, amongst our participants at least, it seemed that this approach often led to shallow exchanges that did not address the deeper emotional needs associated with death and bereavement. The strategies used by young adults to discuss death are likely shaped by broader societal attitudes around reluctance to confront death openly. However, this is not necessarily indicative of death-denial at the societal level (as Kellehear [46] hypothesises) but may rather stem from a lack of confidence at the individual level in navigating potential interpersonal discomfort. Encouragingly, this suggests that through a better understanding of these concerns and strategies used to obscure death-talk, efforts to improve death literacy, as recommended for fostering more open and supportive conversations about mortality, can be successful [47]. Notably, the relationship between death literacy levels and age is not linear, reiterating the need for a life-course approach [48].

Participants identified a range of psychological barriers, including existential anxiety. Existential anxiety, characterised by fear of one’s mortality, further complicated these discussions for young people. Their attempts to avoid acknowledging these fears and vulnerabilities likely serve only to sustain anxiety [49]. Additionally, pervasive negative perceptions of death, influenced by cultural and societal attitudes and narratives, reinforce the perception of death as something to be fearful of and thus, anxiety provoking. These psychological barriers highlight the need for exposure to death literacy through increased public dialogue and educational input to demystify death and address related fears; indeed, recent research does link increasing death-literacy to a reduction in death anxiety [50]. Further, promoting a more open and supportive environment through facilitating safe spaces for communal support and discussion could provide individuals with the opportunity to share these experiences and feelings, reducing the stigma and fear associated with death-talk [51]. Death cafés have been shown to be an acceptable and positive experience for young adult populations such as university students [52, 53]. Greater provision of death cafes and targeted encouragement for younger demographics to participate could help meet this need.

Participants consistently referred to the media’s influence in shaping their perspectives of death-talk. As young adults report a lack of death-talk in their own lives, a significant proportion of their exposure to these discussions likely comes from media coverage, where death is often sensationalised. It was reported that exposure to ‘extreme’ deaths as a result of media preoccupation with such cases impacts public perceptions of death. This saturation around potentially sensationalised and ‘unnatural deaths’ along with a deficiency of coverage of more naturally occurring deaths may further social sequestration of death [54] as death becomes associated with extreme circumstances rather than natural processes. The anxiety felt as a consequence of sociocultural shaping, such as skewed media narratives, is related to avoidance of death-talk [55] and was identified as a barrier by participants. This study and previous research have identified fear as a barrier to death-talk engagement [25, 26, 47]. Collaboration with media outlets, including online/social media platforms, to provide more balanced coverage, such as stories of natural and peaceful deaths, could help shift public perceptions by highlighting positive aspects of end-of-life care and the importance of open dialogue around death.

Resonating with previous work [27] participants in this study reported that successful, meaningful engagement with death-talk is a learned skill that requires guidance and direction rather than being innate. Due to a lack of formal instruction on this, our participants reported feeling ill-equipped to engage in conversations around death and dying. Participants strongly advocated introducing death-talk education at a younger age to normalise these conversations by encouraging honesty and transparency and reducing associated fear and stigma, a shift supported by previous research recommendations [28]. Findings from Islam et al., [27] and Graham-Wisener et al., [9] further underscore the centrality of education as a facilitator of death-talk, with increasing evidence suggesting the importance of creating supportive environments and educational frameworks to help navigate the complexities of bereavement. This approach would involve systemic changes, including the continuous integration of age-appropriate death education throughout school curricula, normalising death discussions, teaching communication and emotional regulation skills and promoting a cultural shift towards accepting and managing emotional conversations. There is evidence that adolescents are willing to discuss death and dying [56] suggesting that clinicians and educators can develop successful programs which engage school age children and young adults in meaningful death-talk and improving their ability to handle these conversations constructively. Whilst this need around death education has been previously recommended [57], shown to be acceptable to children and parents [58] and has begun to be integrated into school curricula, it is at present actioned in isolated areas only [59–61] as interventions are seeking to be developed. Beyond schools, there is encouraging work developing compassionate university campuses, which is directly relevant to the population within this study, but uptake has yet to become universal [62, 63].

As evidenced, this work contributes to a growing body of evidence and opinion which calls for a public health approach to death and dying. An additional benefit arising from facilitating increased death-literate, compassionate communities is the focus this brings to social and health inequalities in access to end-of-life care. Studies indicate that marginalised populations, including racial minorities and low-income groups experience disparities in palliative care and advance care planning [64, 65]. The increase in death-literacy and wider discourse on death may help expedite the creation of policies that address systemic barriers to high-quality end-of-life care. This is increasingly important given the current debates in some countries, including the UK, about legalised assisted dying, where it is essential that any new policies and frameworks represent the ethical position of the fullest spectrum of society which must include its younger members.

### Strengths and limitations

A key strength of this work is its qualitative nature, particularly the use of focus groups, which facilitated in-depth exploration of participants’ attitudes towards discussing death and dying. We considered how to address relevant quality criteria [66] throughout. The study generated a large and rich dataset, capturing the perspectives of young adults from diverse UK regions. This geographic and demographic diversity enhances the transferability and potential impact of the findings. Additionally, the study aligns with criteria such as transparency, rigor and richness by demonstrating thoroughness in data collection and analysis.

Reflexive thematic analysis does not aim for objectivity; instead, it emphasises awareness of how researcher experience shapes their perception of the data [67]. By engaging reflexively, the subjective nature of the researcher’s interpretation is viewed not as a limitation but as an asset to the research [42]. Recruitment, data generation (focus group discussions) and data analysis were led by the first author (CMD). Given her academic background, when interpreting the data the researcher may have been particularly attuned to identifying psychological barriers amongst participants’ accounts, with disciplinary context and training impacting which parts of the data the researcher was drawn to [42]. Additionally, there is the potential for analysis to reflect the researchers’ professional experiences and understanding of effective communication in palliative care settings. Thus, whilst the researcher’s personal and professional experiences provided valuable authenticity, they also necessitated the careful maintenance of a reflexive stance, continually questioning how her positionality might shape the data generation and interpretation and to ensure a comprehensive exploration of diverse perspectives. This was achieved through iterative engagement with a reflexive diary and through ongoing discussion and reflection with other research team members with a variety of different expertise and experience.

The sample size of 33 participants across four focus groups, provides a solid foundation for the study’s findings. It is possible that the reliance on volunteer sampling and virtual focus groups, necessitated by pragmatic considerations, could have introduced selection bias and may not fully capture the experiences of individuals less interested in or comfortable discussing death and dying and/or those less comfortable with technology. However, the use of a maximum variation sampling technique aimed to capture a diverse range of perspectives by considering key characteristics such as education level, age, ethnicity, gender, and religious belief; all identified as influential [28], or under-represented [9, 27] in previous research. This strategy enhanced the robustness of the findings by ensuring that the study considered diverse viewpoints and cultural contexts within the target population. Broad coverage of UK geographical regions was achieved, including participants from all home nations – England, Scotland, Wales and Northern Ireland - suggesting that our findings could be a good indicator of what may be important to other young adults across the UK. At the same time, we fully acknowledge the situated norms of this research, undertaken as it was in a UK setting. International research in other settings is needed to explore the role of culture and societal norms in relation young adults’ attitudes to death talk more fully. Engaging more under-researched groups (e.g., lower socio-economic status, identify as having a religious faith, black ethnicity) in future research would also be beneficial in capturing a wider narrative and confirming suggested intervention strategies or identifying what adaptations might be needed to interventions.

Mapping identified barriers and facilitators onto the COM-B provided a structured approach to understanding behaviour change related to death-talk and is a strength of this work. This application of theory offers a robust a foundation for developing intervention strategies. A theory-driven approach is recommended in future research in order to facilitate the design and implementation of potential intervention approaches, including within education, to enhance death literacy and reduce psychological barriers to death-talk across communities.

## Conclusion

This qualitative study offers novel insights into the complex interplay of psychological, social, and educational factors influencing young adults’ attitudes towards and perceptions of discussing death and dying; a group previously under-represented in this area of research. By drawing on in-depth, rich, contextualised data, this study addresses a gap in the literature and identifies key strategies young adults use to navigate death-talk.

The identification of barriers and facilitators, mapped onto the COM-B framework, provides a structured lens through which to understand the behavioural components that influence engagement in these conversations. Using the COM-B approach was particularly valuable in translating qualitative insights into actionable targets for intervention. It highlighted the need to enhance psychological and social capabilities, expand opportunities for dialogue, and shift motivational attitudes, thereby supporting more effective behaviour change strategies.

To improve death literacy and cultural readiness for such discussions, interventions should focus on early education and media engagement. Integrating age-appropriate death education into school curricula could normalise open dialogue, reduce fear, and build communication and emotional regulation skills. Similarly, more balanced media portrayals may help to reshape public perceptions and reduce avoidance.

By addressing the psychological, social, and environmental factors that shape young adults’ readiness for death-talk, targeted public health strategies can foster more open, meaningful conversations and contribute to a broader cultural shift towards acceptance, preparedness, and public health approaches to palliative care.

## Supplementary Information

Supplementary Material 1.

## Data Availability

The datasets used and analysed during the current study are available from the corresponding author upon reasonable request.
