# Supplementary material for: Adopting the lens of the COM-B behaviour change model to qualitatively explore and understand public health implications of young adults’ attitudes towards death-talk
Source: BMC Public Health. 2025 Oct 2;25:3307. doi: 10.1186/s12889-025-24519-w (PMC12492765; doi:10.1186/s12889-025-24519-w)
Supplement: Supplementary file 1 — Supplementary Material 1. [file 12889_2025_24519_MOESM1_ESM.docx]

**Topic guide:**

*Total focus group discussion time approx. 60 mins max, with intro and closing the group around 1hr 15mins.*

*Introduction to interview:*

Thank the participant for attending and researcher/s to introduce themselves.

Explain these focus groups are about public perceptions of talking about death and dying.

We will be speaking in general and maybe hypothetical terms. We are not asking you to tell us anything sensitive or upsetting about your own experience. However, we realise that this might be a challenging topic for some people – if you need to take a break or stop your participation in the group, as discussed in the information sheet, that’s absolutely fine to do so.

We hope that everyone will feel comfortable to join in and give their opinions and thoughts on the questions asked. There are no right or wrong answers, and we aren’t expecting everyone to have the same opinions. As such, if your experience or ideas are different to someone else, please feel free to speak up – we would love to hear different perspectives!

Equally if someone says something you don’t agree with, please be polite when you respond and treat others how you would like to be treated. It is hoped and expected that participants treat the focus group as a safe space and maintain the privacy and confidentiality of other participants by ensuring that nothing mentioned in the duration of the discussion leaves the focus group or is disclosed to anybody outside of the focus group.

In the instance that two or more participants within the same focus group make it known that they know each other, they will be asked if they feel comfortable continuing with their participation, or if they would prefer to be allocated to an alternative focus group at a later date.

Remind them how long the group is scheduled to last for and that the group will be recorded, and that the audio recording will be deleted once it is transcribed with all personal details removed. As the focus group is taking place on Zoom, tell participants that whilst video engagement is encouraged, it is not a necessity, and it is ultimately their preference. The video element of the zoom recording will be deleted immediately after focus group completion, as it is not necessary for transcription purposes.

Provide the opportunity to ask questions before starting.

Check they are happy to continue? [if yes, start Zoom recording]

*General prompts:*

Why / why not? Please tell me more about that? Please expand on that? What do you mean by that? Who else agrees with that? Does anyone have any different thoughts about that?

1. Do you think that as a society we are comfortable talking about death and dying?
   1. How much do you think we talk about death and dying in the UK?
   2. Do you think people generally feel comfortable talking about death and dying?
      1. Why not?
      2. Why do you think this?
      3. What do you think impacts how comfortable people feel in talking about it? (e.g., including individual factors – religion, own experiences; or lack of).

**[Est 10 mins]**

1. Again in general terms, in your community or social circle, do people discuss death or dying?
   1. What sort of things do you think people might talk about generally, if they were chatting about death and dying (either with you or with others)?
   2. Who might be involved in those conversations?
   3. When might these conversations happen?
   4. What stops these conversations from happening?

**[Est 5-10 mins]**

1. Do you think it’s important for people to talk about death and dying?
   1. In what situations (i.e. day to day or only if someone is elderly/ frail / at end of life)
   2. Are there benefits to talking about death and dying?
      1. What might those benefits be? To whom?

**[Est 5 mins]**

1. Do you think we should try and encourage people to talk more about death and dying?
   1. Why/why not?
   2. If yes: what do you think we could do to encourage people to talk about it more?

**[Est 5 mins]**

1. Do you think perceptions and attitudes around death and dying have changed over time?
   1. What do you think has changed?
   2. What do you think has contributed to these changes?
   3. What impact, if any, do you think the pandemic has had on attitudes, perceptions and discussions around death and dying?
   4. Do you think media depictions/portrayals of death and dying impact how we talk about death and dying? E.g., Adverts/ Gaming/ Films/ Books/ News stories/social media, memorializing - change in public nature of grieving.
      1. Willingness to talk / how often we talk about death and dying?
      2. What we talk about?

**[Est 10-15 mins]**

Thank you for all your thoughts so far. Drawing things to a close now -

Thinking about all of the things we have discussed today:

1. What do you think worries, concerns, or stops people **most** from talking about Death and Dying?
2. What could be done to help people overcome these specific worries/concerns?
   1. Additional probe, if needed: What could help people feel more comfortable / open to conversations about death and dying?

**[Est 10-15 mins]**

CLOSE –

Thank you again so much for your really valuable contributions today. Is there anything else you would like to say about talking about death and dying that we’ve not already spoken about?

**[Est 2-3 mins ]**

[TURN OFF RECORDER, thank everyone again. Notify everyone that they will be emailed a copy of the debrief sheet via email.]
